# Reliability and validity of the Turkish EQ-5D-5L in adults living with HIV

**DOI:** 10.7717/peerj.21727

**Published:** 2026-09-12

**Authors:** Recep Balık, Nurgül Ceran

**Affiliations:** Department of Infectious Diseases and Clinical Microbiology, University of Health Sciences Haydarpaşa Numune Training and Research Hospital, Istanbul, Turkey

**Keywords:** EQ-5D-5L, PLHIV, Health-related quality of life, Reliability, Validity

## Abstract

**Background:**

The advent of antiretroviral therapy (ART) has shifted human immunodeficiency virus (HIV) care from survival toward optimizing quality of life. In the contemporary ART era, health-related quality of life (HRQoL) is recognized as a core outcome in HIV care. The EQ-5D-5L is widely used to measure HRQoL. However, its measurement properties have not been evaluated in Turkish people living with HIV (PLHIV). This study aimed to evaluate the reliability and validity of the Turkish EQ-5D-5L in PLHIV.

**Methods:**

We conducted a psychometric validation study at a tertiary center and enrolled 157 PLHIV. For test-retest reliability, 60 participants completed the EQ-5D-5L on two occasions. Because Turkey currently lacks an EQ-5D-5L value set, and to enhance the robustness of interpretation , utility scores were calculated using five international value sets. We assessed internal consistency, test-retest reliability, internal structure using confirmatory factor analysis (CFA), and construct validity using known-groups comparisons and within-instrument convergent and discriminant validity analyses.

**Results:**

The cohort had a median age of 41 years, was predominantly male (93%), mostly virally suppressed (94.3%), and had generally favorable immunological status. A ceiling effect was observed in 36.9% of participants. Internal consistency was high (ordinal α = 0.930; ω total = 0.954). Test-retest reliability was good for utility indices (intraclass correlation coefficient (ICC) = 0.799–0.886) and the level sum score (ICC = 0.880), but lower for the EuroQol Visual Analogue Scale (EQ-VAS) (ICC = 0.656). Model fit was good (Comparative fit index (CFI) = 0.999; root mean square error of approximation (RMSEA) = 0.053), and standardized loadings ranged from 0.651 (for anxiety/depression) to 0.975 (for usual activities). Known-groups comparisons showed significantly lower utility scores and EQ-VAS values in participants with comorbidities (*p* < 0.05). Within-instrument convergent validity was supported by moderate correlations between utility indices and EQ-VAS (ρ = 0.602–0.615).

**Conclusion:**

The Turkish EQ-5D-5L demonstrates acceptable reliability and construct validity in PLHIV receiving routine care, and CFA suggested a coherent internal response structure in this sample. Ceiling effects in this clinically stable cohort warrant consideration when selecting outcomes for studies targeting subtle within-person change. Development of a Turkey-specific value set remains a priority.

## Introduction

Human immunodeficiency virus (HIV) infection remains a major global public health problem. In 2024, an estimated 40.8 million people were living with HIV globally, with approximately 1.3 million new infections and 630,000 Acquired immunodeficiency syndrome (AIDS)-related deaths (World Health Organization, 2025). With the advent of antiretroviral therapy (ART), HIV is increasingly managed as a long-term condition in settings where lifelong treatment is available, shifting attention toward outcomes beyond virological control alone (Deeks, Lewin & Havlir, 2013). Health-related quality of life (HRQoL) is therefore increasingly treated as a core outcome in HIV care because clinical endpoints do not fully capture day-to-day functioning and well-being (Cooper et al., 2017). In 2016, Lazarus and colleagues proposed extending the UNAIDS 90–90–90 treatment cascade with a fourth “90” that explicitly targets HRQoL among individuals with viral suppression (Lazarus et al., 2016; Webster, 2019). This additional target implies that 90% of people living with HIV (PLHIV) who attain viral load suppression should also report good HRQoL, thereby elevating HRQoL to a patient-centered outcome alongside viral suppression (The Lancet HIV, 2019; Kall et al., 2020). Separately, the cascade goals were later revised upward to 95–95–95; in the 2021 UN General Assembly Political Declaration on HIV and AIDS in which member States committed to achieving by 2025: 95% of all people living with HIV knowing their status, 95% of those diagnosed receiving antiretroviral therapy, and 95% of those on treatment attaining viral suppression (UNAIDS, 2026a; UNAIDS, 2026b).

The EQ-5D-5L is a generic, preference-based HRQoL instrument developed by the EuroQol Group. It describes health across five dimensions with five response levels, producing standardized health profiles and utility indices for comparative and health-economic analyses (Herdman et al., 2011).

Internationally, the EQ-5D family has been used as a brief generic HRQoL instrument in PLHIV, and systematic-review evidence supports EQ-5D among generic measures used in HIV research; however, EQ-5D-5L-specific psychometric evidence in PLHIV is still comparatively limited when compared with longer HIV-specific instruments such as The Medical Outcomes Study HIV Health Survey (MOS-HIV) or World Health Organization Quality of Life-HIV instrument, Brief version (WHOQoL-HIV-BREF) (Cooper et al., 2017; Wen et al., 2022). In Turkey, an official Turkish EQ-5D-5L version is available through EuroQol, but published evidence on its measurement properties remains limited. Outside HIV, the main Turkish clinical psychometric evidence comes from patients undergoing coronary artery bypass graft surgery (Köken et al., 2024). The largest HIV-specific EQ-5D-5L psychometric study, conducted among 1,016 ART-clinic patients in Vietnam, reported acceptable internal consistency, convergence with Visual Analogue Scale (VAS), and discrimination by HIV/AIDS stage, CD4 count, and ART duration (Tran, Ohinmaa & Nguyen, 2012). PLHIV may have a different HRQoL profile from other clinical populations: in the ART era, many patients are virologically suppressed and physically stable, which can produce high utility scores and ceiling effects, while mental-health problems may remain prominent. In large EQ-5D-5L ART-era cohorts, anxiety/depression was the most affected dimension despite relatively high overall utility values (Popping et al., 2021). Taken together, these findings indicate that the Turkish EQ-5D-5L cannot be assumed to perform similarly in PLHIV without population-specific validation. Accordingly, this study aimed to evaluate the reliability and validity of the Turkish EQ-5D-5L in PLHIV, following Consensus-based Standards for the selection of health Measurement Instruments (COSMIN) reporting guidance for measurement-property studies (Gagnier et al., 2021; Mokkink et al., 2018).

## Materials & Methods

### Study design and participants

This cross-sectional psychometric validation study was conducted at Haydarpaşa Numune Training and Research Hospital, a tertiary referral center in Istanbul, Turkey, between March and August 2025. The study evaluated the reliability, validity, and other measurement properties of the Turkish version of the EQ-5D-5L in adults living with HIV. Eligible participants were adults (≥18 years) with a confirmed HIV diagnosis and sufficient Turkish literacy to complete the questionnaires. During the study period, 172 adults living with HIV attending routine outpatient visits were approached and assessed for eligibility. All 172 met the eligibility criteria; none was excluded because of insufficient Turkish literacy, cognitive impairment, or severe acute illness. Fifteen patients declined participation, and 157 eligible consenting participants were enrolled and included in the final analysis.

*Outcome measures and covariates*:

The primary measurement outcomes were EQ-5D-5L utility indices derived from the selected value sets, the level sum score (LSS), EuroQol Visual Analogue Scale (EQ-VAS), and responses to the five EQ-5D-5L dimensions. Psychometric outcomes included internal-consistency coefficients, test–retest reliability estimates, measurement-error indices, CFA fit indices and factor loadings, and known-groups, convergent, and discriminant validity results. Sociodemographic covariates were age, sex, and educational attainment. Age was analyzed as a continuous variable. Sex was categorized as male or female. Educational attainment was categorized as primary school, high school, or university. Clinical covariates were CD4 cell count, CD4 percentage, time since HIV diagnosis, ART duration, comorbidity status, detectable viral load, and history of AIDS-defining conditions. CD4 cell count, CD4 percentage, time since HIV diagnosis, and ART duration were analyzed as continuous variables. Comorbidity status was categorized as absent or present and was defined as the presence of at least one chronic condition other than HIV infection. Viral load status was categorized as undetectable or detectable; detectable viral load was defined as HIV-1 RNA > 50 copies/mL. History of AIDS-defining conditions was categorized as absent or present and classified according to the Centers for Disease Control and Prevention (CDC) (2014) criteria.

### Instrument

The Turkish EQ-5D-5L was developed by the EuroQol Group following its standardized translation protocol, including independent forward and back translation, expert-panel reconciliation (EuroQol, 2026). Because an officially translated and authorized Turkish version was already available, a de novo translation was not undertaken. However, as the original translation work was conducted in the general population and did not specifically include people living with HIV, we conducted additional cognitive debriefing interviews with the first 20 consecutively recruited adults living with HIV at our center to assess item comprehensibility and cultural appropriateness in this clinical population. Using a standardized cognitive interviewing procedure, interviews combined concurrent think-aloud methods with targeted verbal probes to detect ambiguous wording, response difficulties, and potential cultural or conceptual nonequivalence (Willis, 2015). For each item, participants were asked to explain how they understood the question and how they selected their response level. After probing, participants also rated the perceived clarity of each item on a 5-point scale, where 1 indicated “not clear at all” and 5 indicated “completely clear”. Items were prespecified as acceptable when at least 80% of participants rated them as clear or completely clear. Interviewers documented all comments and probe responses. All items met the ≥80% perceived-clarity criterion and no recurrent comprehension problems or culturally discordant interpretations emerged during probing. Therefore, the Turkish item wording was retained without modification. All questionnaires were reviewed after completion to ensure completeness. The 20 participants who completed cognitive debriefing were included in the final analytic sample because they met the same eligibility criteria as the other participants, completed the same authorized Turkish EQ-5D-5L version, and no item wording was modified after the debriefing procedure. The EQ-5D-5L comprises five dimensions—mobility, self-care, usual activities, pain/discomfort, and anxiety/depression—each rated on a five-level ordinal response scale ranging from no problems to extreme problems; respondents also rate their overall health on a 0–100 vertical VAS (EQ-VAS) (Herdman et al., 2011).

At the time of analysis, a Turkish EQ-5D-5L value set was not available. In line with EuroQol guidance recommending proxy value sets that most closely approximate the target country in sociodemographic, cultural, and health-system characteristics, we prespecified a sensitivity framework using five international tariffs selected to represent distinct societal preference patterns relevant to Turkey’s transcontinental position.

We selected EQ-5D-5L value sets to reflect principal dimension-weighting patterns described in comparative valuation studies. Germany anchors the Western European preference pattern, in which pain/discomfort and anxiety/depression receive the largest decrements (Ludwig, Graf von der Schulenburg & Greiner, 2018). Egypt represents a mobility-dominant pattern described in Middle Eastern and North African (MENA) populations, where physical functioning carries the greatest weight—reflecting Turkey’s geographic and cultural proximity to the MENA region (Al Shabasy et al., 2022). Hungary provides an Eastern European variant in which mobility also ranks highest but is followed closely by pain/discomfort, bridging the Western and mobility-dominant patterns and reflecting Turkey’s socioeconomic and regional ties to Central/Eastern Europe (Rencz et al., 2020). Morocco was included as a second MENA-region tariff to assess the stability of findings within the mobility-dominant pattern and to provide geographic triangulation with Egypt (Azizi et al., 2025). In addition, we applied the UK EQ-5D-3L crosswalk (Van Hout et al., 2012), a widely used interim scoring method when a country-specific 5L tariff is unavailable, to facilitate comparability with studies that have employed this approach. To provide a tariff-independent measure of overall health-state severity, we calculated the LSS by summing response levels across the five EQ-5D-5L dimensions. Total scores range from 5 to 25, with higher scores indicating worse health status. The LSS was analyzed continuously; no cut-off values were applied. Utility indices and LSS were computed using the eq5d R package, which implements published scoring algorithms and value set calculators (Morton & Nijjar, 2025).

### Statistical analysis

All statistical analyses were conducted using R version 4.5.1 (R Core Team, 2025). Continuous variables were summarized as medians and interquartile ranges (Q1–Q3), whereas categorical variables were summarized as frequencies and percentages. Continuous variables were compared between groups using the Mann–Whitney U test.

Ordinal alpha was computed using polychoric correlations among the five dimensions. McDonald’s omega total was calculated from the one-factor CFA model as a descriptive composite estimate under a unidimensional summary model. Cronbach’s alpha was also reported descriptively. Corrected item-total correlations were calculated for each dimension, and alpha-if-item-deleted statistics were used to evaluate the impact of removing individual dimensions on overall reliability.

To assess test–retest reliability, 60 participants were randomly selected from the full study sample after completion of the baseline EQ-5D-5L assessment. Contemporary recommendations for reliability and measurement-error studies emphasize design-specific precision rather than a fixed percentage rule. With 60 participants and two repeated measurements, a planning approximation assuming an ICC of approximately 0.80 indicated an expected 95% confidence-interval width of about 0.19 for the ICC estimate, which was considered acceptable for the primary utility-index reproducibility analyses (Mokkink et al., 2023). Participants completed the EQ-5D-5L a second time after approximately two weeks; the observed mean retest interval was 13.2 ± 1.69 days. At the second assessment, participants were asked whether they perceived any important change in their general health since the initial visit. Test–retest reliability was estimated only among participants who reported no perceived health change between assessments, because reproducibility should be evaluated in participants whose underlying health status is stable. None of the participants reported perceived health change. No retest variables were missing. Test-retest reproducibility for utility scores, LSS, and EQ-VAS was assessed using the two-way random-effects intraclass correlation coefficient for absolute agreement, single measures (ICC[2,1]), with 95% confidence intervals. For the five EQ-5D-5L dimensions, test-retest agreement was evaluated using quadratic weighted Cohen’s kappa with 95% confidence intervals. Exact agreement and within-one-level agreement were also calculated. Measurement error was quantified using the standard error of measurement (SEM) and the smallest detectable change (SDC).

The five EQ-5D-5L dimensions were treated as ordinal descriptive attributes of health status rather than as interchangeable reflective indicators of a single latent HRQoL construct. Accordingly, CFA was used pragmatically to examine the coherence of response patterns among the five dimensions in this sample, not to validate a unidimensional latent construct underlying the EQ-5D-5L. Models were estimated using weighted least squares mean- and variance-adjusted estimation (WLSMV). Model fit was evaluated using the CFI, Tucker-Lewis index (TLI), RMSEA with 90% confidence intervals, and standardized root mean square residual (SRMR). We prespecified a parsimonious one-factor model as a summary representation and estimated a secondary two-factor model separating physical functioning (mobility, self-care, usual activities) and psychosocial dimensions (pain/discomfort, anxiety/depression) for structural comparison, consistent with prior non–preference-based investigations of EQ-5D internal structure (Feng et al., 2019). Nested model comparison was conducted using the scaled chi-square difference test (Satorra, 2000).

Construct validity was evaluated using known-groups comparisons and within-instrument convergent/discriminant validity analyses. In the known-groups analysis, we hypothesized that participants with comorbidities would have worse HRQoL than those without comorbidities. Utility scores, LSS, and EQ-VAS were compared across clinically defined groups expected to differ in health status. Within-instrument convergent validity was assessed using Spearman rank correlations to examine associations among utility scores, LSS, and EQ-VAS. Discriminant validity was evaluated by examining correlations between EQ-5D-5L indices and variables (months since HIV diagnosis and ART duration) not expected to be strongly related to current health status.

### Ethical considerations

The study was approved by the institutional review board (Numune Ethical Review Board; HNEAH-GOAEK 2025/83) and was conducted in accordance with the principles of the Declaration of Helsinki. All participants provided informed written consent prior to enrollment.

The authors used the ChatGPT (GPT-5.1) model to assist with minor English-language editing of the final draft.

## Results

A total of 157 participants were included in the study, and all clinical variables and questionnaire items were complete. Participant characteristics are summarized in Table 1. The median age of the study population was 41.0 (33–49) years. The cohort was largely male (93.0%), with women accounting for 7.0%. Educational attainment was relatively high: 63.1% had university education, 26.8% completed high school, and 10.2% had primary education. Viral suppression was common, with 94.3% having an undetectable viral load. A prior AIDS-defining condition was reported by 3.2%, and 33.1% had at least one comorbid condition. Median time since HIV diagnosis was 83 (56-114) months, and median ART duration was 81 (55–113) months. Immune status was generally favorable (median CD4 826 cells/µL (549–1,051); median CD4% 35.0% (27.8–41.3)).

**Table 1:** Sociodemographic and HIV-related clinical characteristics of the study participants. Baseline participant characteristics for the full study sample (*n* = 157), including demographic variables and HIV clinical indicators. Categorical variables are reported as n (%), and continuous variables as median (Q1–Q3).

Variable	Value
Age (years)	41.0 [33.0–49.0]
Gender	
Male	146 (93.0%)
Female	11 (7.0%)
Education level	
Primary	16 (10.2%)
High school	42 (26.8%)
University	99 (63.1%)
Undetectable viral load	148 (94.3%)
AIDS-defining condition history	5 (3.2%)
Comorbid condition ≥1	52 (33.1%)
HIV duration (months)	83 [56–114]
ART duration (months)	81 [55–113]
CD4 count (cells/µL)	826 [549–1051]
CD4 percentage (%)	35.0 [27.8–41.3]

**Notes.**

AIDS, acquired immunodeficiency syndrome; ART, antiretroviral therapy; HIV, human immunodeficiency virus.

Across EQ-5D-5L dimensions, responses clustered at Level 1 in the physical domains, whereas anxiety/depression showed a greater burden of reported problems (Table 2). Level 1 responses were most frequent for mobility (80.3%), self-care (86.6%), and usual activities (73.9%), with relatively few participants endorsing severe or extreme problems (Levels 4–5) in these domains (mobility: 1.9%; self-care: 1.2%; usual activities: 1.9%). Pain/discomfort showed a broader spread, with more responses at Levels 2–3 than other physical dimensions, despite Level 1 remaining most common. Anxiety/depression had the widest distribution: 42.0% reported Level 1, while 58.0% reported at least some anxiety/depression. Moderate-to-extreme problems (Levels 3–5) were reported by 31.8%, and severe or extreme problems (Levels 4–5) by 8.9% (Table S1).

**Table 2:** Distribution of EQ-5D-5L responses by dimension and severity level (n, %). Baseline EQ-5D-5L profile showing the number and percentage of participants selecting each severity level (1–5) within each of the five EQ-5D-5L dimensions.

Dimension	Level 1	Level 2	Level 3	Level 4	Level 5
Mobility	126 (80.3)	18 (11.5)	10 (6.4)	2 (1.3)	1 (0.6)
Self-care	136 (86.6)	15 (9.6)	4 (2.5)	1 (0.6)	1 (0.6)
Usual activities	116 (73.9)	28 (17.8)	10 (6.4)	2 (1.3)	1 (0.6)
Pain/discomfort	110 (70.1)	33 (21.0)	10 (6.4)	3 (1.9)	1 (0.6)
Anxiety/depression	66 (42.0)	41 (26.1)	36 (22.9)	12 (7.6)	2 (1.3)

**Notes.**

Values are n (%).

Inter-item correlation coefficients ranged from 0.312 to 0.755. The strongest correlation was observed between usual activities and pain/discomfort (*r* = 0.755), followed by usual activities and mobility (*r* = 0.713). Mobility showed moderate correlations with pain/discomfort (*r* = 0.633) and self-care (*r* = 0.576). The anxiety/depression dimension demonstrated comparatively lower correlations with the other dimensions, ranging from 0.312 (self-care) to 0.505 (pain/discomfort). The correlation between anxiety/depression and usual activities was 0.441, and with mobility was 0.388. Overall, correlations were higher among the physical-functioning dimensions, while anxiety/depression showed weaker associations with the remaining domains.

Median utility values were high across all value sets (Table 3). The UK 3L crosswalk produced a median utility of 0.848, while median utilities for the 5L tariffs were 0.970 for Germany, 0.960 for Hungary, 0.946 for Egypt, and 0.964 for Morocco. The median EQ-VAS score was 88.0, and the LSS had a median of 6.00. Negative utility values were infrequent across tariffs, occurring in 3.8% of participants with the UK 3L crosswalk, 0.6% with the Germany and Hungary 5L tariffs, 1.9% with the Egypt 5L tariff, and 1.3% with the Morocco 5L tariff. Utility indices were highly correlated across value sets and showed the expected inverse correlation with the LSS (Table S2).

**Table 3:** EQ-5D-5L utility indices, EQ-VAS, and level sum score. Summary of baseline health outcomes expressed as EQ-5D-5L utility indices calculated using multiple scoring algorithms (UK 3L crosswalk; Germany, Hungary, Egypt, and Morocco 5L value sets), alongside EQ-VAS and the level sum score (LSS).

Measure	Median [Q1–Q3]	Participants with negative utility values, n (%).
UK 3L crosswalk	0.848 [0.689–1.000]	6 (3.8%)
Germany 5L	0.970 [0.851–1.000]	1 (0.6%)
Hungary 5L	0.960 [0.847–1.000]	1 (0.6%)
Egypt 5L	0.946 [0.765–1.000]	3 (1.9%)
Morocco 5L	0.964 [0.835–1.000]	2 (1.3%)
EQ-VAS	88.0 [75.0–96.0]	–
Level sum score	6.00 [5.00–8.00]	–

**Notes.**

Values are median (Q1–Q3). Negative utilities are n (%), applicable to utility indices only. EQ-VAS, EuroQol Visual Analogue Scale.

Internal consistency was evaluated using reliability coefficients appropriate for ordinal EQ-5D-5L responses. Ordinal alpha (polychoric) was 0.930, McDonald’s omega was 0.954, and conventional Cronbach’s alpha was 0.843. Corrected item–total correlations ranged from 0.491 (anxiety/depression) to 0.808 (usual activities) (Table S3).

Test–retest reproducibility analyses showed that utility indices demonstrated good reproducibility across tariffs, with ICC values ranging from 0.799 to 0.886. The highest reproducibility was observed for the Egypt 5L tariff, whereas the UK 3L crosswalk showed the lowest ICC among utility measures. The LSS also showed high reproducibility, while EQ-VAS demonstrated comparatively lower reproducibility. SEM and SDC were small for the utility indices relative to their scale range, whereas larger values were observed for EQ-VAS. Bland–Altman analyses showed only small mean differences across measures; bias tests were not significant, suggesting no systematic shift between test and retest. Limits of agreement were tighter for the direct 5L tariffs than for the UK 3L crosswalk and widest for EQ-VAS (Table 4). At the dimension level, agreement was moderate to excellent: weighted kappa ranged from 0.422 (self-care) to 0.844 (mobility). Exact agreement was 70.0%–90.0%, and agreement within one response level was consistently high—often 100% and always above 95% across dimensions (Table 5).

**Table 4:** Test–retest reproducibility, measurement error, and Bland–Altman agreement for EQ-5D-5L utilities, level sum score, and EQ-VAS. Test–retest agreement for EQ-5D-5L utility indices (by tariff), LSS, and EQ-VAS in the retest subsample (*n* = 60). The bias *p*-value tests whether the mean difference deviates from zero.

Measure	ICC (95% CI)	SEM	SDC	Bland–Altman agreement (Mean difference (Lower LOA, Upper LOA))	Bias *p*-value
UK 3L crosswalk	0.799 (0.685–0.875)	0.113	0.314	0.032 (−0.252–0.316)	0.096
Germany 5L	0.828 (0.728–0.894)	0.058	0.161	0.015 (−0.133–0.164)	0.120
Hungary 5L	0.877 (0.801–0.925)	0.049	0.135	0.016 (−0.112-0.143)	0.069
Egypt 5L	0.886 (0.816–0.930)	0.067	0.186	0.017 (−0.163–0.198)	0.151
Morocco 5L	0.841 (0.748–0.902)	0.077	0.214	0.015 (−0.190–0.219)	0.280
Level sum score (LSS)	0.880 (0.807–0.926)	0.902	2.499	−0.183 (−2.670–2.304)	0.268
EQ-VAS	0.656 (0.486–0.779)	11.144	30.889	2.083 (−25.570–29.737)	0.257

**Notes.**

EQ-VAS, EuroQol Visual Analogue Scale; ICC, intraclass correlation coefficient (two-way random effects, absolute agreement); SEM, standard error of measurement; SDC, smallest detectable change; LSS, level sum score; LOA, limits of agreement. Values are based on the test–retest subsample (*n* = 60).

**Table 5:** Quadratic weighted kappa (95% CI) and agreement rates by EQ-5D-5L dimension. Item-level (dimension-level) test–retest agreement for EQ-5D-5L responses in the retest subsample (n=60), summarized using quadratic weighted Cohen’s kappa (with 95% CI), exact agreement (% identical levels), and agreement within ±1 response level (%)

Dimension	Weighted *κ*	95% CI	Exact agreement (%)	Within one level (%)
Mobility	0.844	0.709–0.979	90.0	100.0
Self-care	0.422	0.122–0.722	83.3	100.0
Usual activities	0.830	0.715–0.945	88.3	100.0
Pain/discomfort	0.668	0.527–0.809	70.0	98.3
Anxiety/depression	0.841	0.736–0.945	75.0	96.7

**Notes.**

*κ*, quadratic weighted Cohen’s kappa; CI, confidence interval.

Internal structure was evaluated using confirmatory factor analysis (CFA). The prespecified single-factor model demonstrated good fit to the data (*χ*^2^ (5) = 7.169, *p* = 0.208; CFI = 0.999; TLI = 0.998; RMSEA = 0.053, 90% CI [0.000–0.085]; SRMR = 0.030). Standardized factor loadings ranged from 0.651 to 0.975, with the highest loading observed for usual activities and the lowest for anxiety/depression; item-level explained variances (R^2^) ranged from 0.424 to 0.950 (Table 6).

**Table 6:** Confirmatory factor analysis results for the EQ-5D-5L dimensions: standardized factor loadings and explained variance. Standardized factor loadings and explained variance (*R*^2^) for each EQ-5D-5L dimension under the prespecified single-factor CFA model. Loadings quantify the strength of association between each dimension and the latent factor.

Dimension	Standardized loading	*R* ^2^
Mobility	0.888	0.789
Self-care	0.861	0.741
Usual activities	0.975	0.950
Pain/discomfort	0.908	0.825
Anxiety/depression	0.651	0.424

**Notes.**

Loadings are standardized. R^2^ is the proportion of variance in each dimension explained by the latent factor. CFA, confirmatory factor analysis.

A secondary two-factor model separating physical functioning (mobility, self-care, usual activities) and psychosocial dimensions (pain/discomfort, anxiety/depression) was also estimated. This model showed similarly good fit (*χ*^2^ (4) = 4.921, *p* = 0.295; CFI = 1.000; TLI = 0.999; RMSEA = 0.038, 90% CI [0.000–0.080]; SRMR = 0.021). However, the improvement in fit relative to the single-factor model was not statistically significant (scaled Δ*χ*^2^ = 1.957, Δ*df* = 1, *p* = 0.162). Considering the comparable fit indices and the intended use of the EQ-5D-5L as a single utility-based measure, the one-factor model was retained as the primary parsimonious summary of the covariance structure.

Known-groups comparisons supported construct validity: participants with ≥1 comorbid condition had lower median utility scores across all tariffs and lower EQ-VAS, while LSS was higher (worse) in the comorbidity group. Effect sizes were positive for utilities and EQ-VAS (higher scores in the no-comorbidity group), whereas the LSS effect size was negative because higher LSS indicates worse health (Table 7). Comparisons involving rare or highly imbalanced subgroups (history of an AIDS-defining condition, *n* = 5; detectable viral load, *n* = 9) were underpowered and are summarized as exploratory in Table S4.

**Table 7:** Known-groups comparison of EQ-5D-5L utility scores, level sum score, and EQ-VAS by comorbidity status, including *p*-values and effect sizes. Comparison of EQ-5D-5L utility indices (by tariff), LSS, and EQ-VAS between participants without comorbidity (*n* = 105) and with ≥1 comorbid condition (*n* = 52).

Outcome	No comorbidity (*n* = 105)	Comorbidity (*n* = 52)	*p*-value	Rank-biserial r
UK 3L	0.848 [0.744–1.000]	0.788 [0.594–1.000]	0.006	0.361
Germany 5L	0.970 [0.913–1.000]	0.918 [0.800–1.000]	0.010	0.296
Hungary 5L	0.960 [0.907–1.000]	0.907 [0.789–1.000]	0.008	0.338
Egypt 5L	0.946 [0.819–1.000]	0.841 [0.639–1.000]	0.015	0.379
Morocco 5L	0.964 [0.841–1.000]	0.897 [0.750–1.000]	0.015	0.298
Level sum score	6.0 [5.0–7.0]	7.00 [5.0–10.0]	0.006	−0.477
EQ-VAS	90.0 [80.0–97.0]	81.5 [70.0–95.0]	0.017	0.409

**Notes.**

Values are median [Q1–Q3]. Mann–Whitney U test (two-sided). Effect size is rank-biserial correlation computed using the group order shown. Positive values indicate higher outcome values in the no-comorbidity group; for LSS, a negative effect size indicates worse LSS in the comorbidity group. LSS, level sum score; EQ-VAS, EuroQol Visual Analogue Scale.

An exploratory sensitivity power calculation was performed only to contextualize the magnitude of effects detectable in the primary known-groups comparison. With group sizes of 105 and 52 and a two-sided *α* of 0.05, the available sample had 80% power to detect an effect size of Cohen’s *d* = 0.48, approximately equivalent to rank-biserial correlation of *r* ≈ 0.23.

Within-instrument convergent validity was supported by strong correlations among utility indices derived from different value sets (ρ = 0.959–0.995, *p* < 0.001). Utility scores showed strong inverse correlations with the LSS (ρ = −0.973 to −0.992, *p* < 0.001). Moderate correlations were observed between EQ-VAS and utility indices (ρ = 0.602–0.615, *p* < 0.001), and EQ-VAS showed a moderate negative correlation with LSS (ρ = −0.614, *p* < 0.001), consistent with the expected direction of scoring (Table S2).

Within-instrument discriminant validity was supported by near-zero correlations between EQ-5D-5L indices and variables not expected to reflect current health status. Correlations between utility scores and time since HIV diagnosis, and ART duration were small (ρ values approximately −0.060 to 0.010). Similarly, LSS and EQ-VAS showed negligible associations with these variables (ρ = −0.135 to 0.047). As expected, duration since diagnosis and ART duration were strongly correlated with each other (*ρ* = 0.998, *p* < 0.001).

## Discussion

This study evaluated the psychometric performance of the Turkish EQ-5D-5L in PLHIV receiving routine outpatient care. Overall, the findings support acceptable measurement performance: reproducibility of utility indices was good across tariffs, dimension-level agreement was moderate to excellent, CFA showed good fit with differential loadings across dimensions, and construct validity was supported by expected known-groups differences by comorbidity and by within-instrument associations with EQ-VAS. The cohort’s long-standing ART use, high viral suppression, favorable immune status, and low prevalence of AIDS-defining conditions provide a plausible explanation for the generally high utility and EQ-VAS values observed (Cooper et al., 2017; Popping et al., 2021).

A key descriptive finding was the concentration of responses at the best level for the physical-functioning dimensions, with 36.9% of participants reporting the best possible health state (11111). This exceeds the ≥15% threshold commonly cited as indicative of a problematic ceiling effect (Cheng et al., 2024) and is consistent with a pooled ceiling proportion of 31.5% reported in a recent meta-analysis of EQ-5D-5L general-population surveys (Cheng et al., 2024). Importantly, the observed ceiling effect is plausibly attributable to the health distribution of this clinically stable cohort rather than a definitive limitation of the Turkish wording. Ceiling effects reduce discrimination among individuals at the healthier end and may attenuate responsiveness to subtle within-person change in longitudinal contexts (Tran, Ohinmaa & Nguyen, 2012). In our cohort, anxiety/depression showed the widest spread of responses and a higher proportion of moderate-to-severe problems than the physical dimensions (Table 2). Similar patterns in PLHIV—where anxiety/depression remains salient despite otherwise favorable overall profiles—have been reported in ART-era studies and syntheses, supporting the clinical relevance of these dimensions for HRQoL profiling (Popping et al., 2021; Miners et al., 2014; Zhou et al., 2021).

Because Turkey currently lacks a country-specific EQ-5D-5L value set, utilities were derived using multiple international tariffs. This improves transparency and highlights tariff dependence: although the overall pattern of high health status was consistent, absolute utility values varied by value set, and the UK 3L crosswalk yielded comparatively lower utilities and a greater frequency of negative values than the direct 5L tariffs. These findings underscore that absolute utilities are not directly comparable across studies unless the same valuation system is used, and they motivate the development of a Turkey-specific EQ-5D-5L value set to support nationally anchored economic evaluation and outcomes research (Tran, Ohinmaa & Nguyen, 2012; Cheng et al., 2024; Belay et al., 2021; Owusu et al., 2024).

Internal consistency was high: ordinal alpha (0.93) and omega total (0.954) exceeded commonly cited benchmarks for group-level research. Traditional Cronbach’s alpha (0.843) was lower, which is expected with ordered categorical items because Pearson-based correlations can underestimate underlying associations—particularly when response distributions are skewed and response options are few—whereas ordinal alpha derived from polychoric correlations is recommended as the primary estimate for ordinal scales (Gadermann, Guhn & Zumbo, 2012). Cronbach’s alpha in our study is comparable to prior EQ-5D-5L applications in PLHIV and other populations (Tran, Ohinmaa & Nguyen, 2012; Xu et al., 2022; Bilbao et al., 2018). Nevertheless, the ordinal coefficients were notably high for a five-item instrument. Several inter-item correlations among the physical-functioning dimensions exceeded 0.70 (*e.g.*, usual activities–pain/discomfort, *r* = 0.755; usual activities–mobility, *r* = 0.713), suggesting that in this clinically stable cohort with pronounced ceiling effects, limitations in these domains tend to co-occur and may offer limited discriminant content at the healthier end of the spectrum. Conversely, the lower corrected item–total correlation for anxiety/depression (0.491 *vs.* 0.621–0.808 for physical dimensions) is consistent with the CFA loading pattern, supporting the interpretation that psychological distress is partially distinct from the general health factor in this population.

Test-retest findings indicated good reproducibility for preference-based utility indices across tariffs over the two-week interval, whereas EQ-VAS reproducibility was lower. The wide EQ-VAS limits of agreement indicate that test–retest differences of up to 30 points on a 0–100 scale may occur in the absence of true change, suggesting that EQ-VAS may have limited value for individual-level monitoring in this population and is better suited for group-level comparisons where random error averages out (Cheng, Tan & Luo, 2021). Among the utility indices, the UK 3L crosswalk showed wider limits of agreement (−0.252 to +0.316) than the direct 5L tariffs, consistent with the additional measurement uncertainty introduced by the crosswalk mapping algorithm. These findings support preferential use of direct 5L value sets over the 3L crosswalk when test–retest precision is important (Van Hout et al., 2012).

At the dimension level, agreement was substantial to almost perfect for most domains. The lower kappa coefficient for self-care despite high absolute agreement is plausibly attributable to marginal distribution effects in a ceiling-skewed item, where limited variability can reduce chance-corrected agreement. Measurement error estimates (SEM/SDC) help interpret within-person differences attributable to measurement error (De Vet et al., 2011; Whynes & the TOMBOLA Group, 2008).

CFA indicated that the five EQ-5D-5L dimension responses shared substantial variance in this cohort, with higher loadings for physical-functioning dimensions and a lower loading for anxiety/depression. Given the EQ-5D-5L’s descriptive, preference-based design, these findings should be interpreted as describing internal response coherence in this sample rather than demonstrating a unidimensional latent HRQoL construct. The comparatively lower anxiety/depression loading supports examining psychological distress at the domain level in addition to summary indices (Feng et al., 2019). This pattern was also reflected in the inter-dimension correlations, where anxiety/depression showed the weakest association with self-care, suggesting that psychological distress and basic self-care capacity are largely independent in this clinically stable cohort.

Known-groups validity was supported by consistent differences by comorbidity status across utilities, LSS, and EQ-VAS, indicating discrimination between clinically plausible groups in the expected direction. Convergent validity was supported by moderate associations between EQ-VAS and utility indices, consistent with the expectation that global self-rated health and preference-based utilities are related but not interchangeable constructs. Conversely, correlations between HRQoL outcomes and years since diagnosis or ART duration were near zero, as expected in a cross-sectional outpatient cohort with high viral suppression and favorable immune status, where duration measures are not direct proxies for current symptoms, functioning, or psychological well-being. Subgroup analyses by AIDS-defining condition history and detectable viral load were underpowered in this sample and should not be interpreted as evidence of no association (Miners et al., 2014; Van Duin et al., 2017; Xu et al., 2024).

Strengths include the use of psychometric methods suited to EQ-5D-5L data, including reliability coefficients appropriate for ordinal indicators, explicit evaluation of agreement and measurement error, and internal structure assessment using robust estimation. Reporting results across multiple international value sets is transparent and defensible while a Turkey-specific EQ-5D-5L tariff is unavailable. The main limitation is the marked ceiling effect in this clinically stable cohort, which compresses scores at the healthier end, limits discrimination among individuals with good health, and likely reduces sensitivity to small within-person changes over time. Because EQ-5D-5L dimensions are conceptually distinct attributes used to define health states for preference-based scoring, internal-consistency indices and CFA should be interpreted cautiously and were used here primarily to describe response coherence rather than to assert unidimensional measurement. Generalizability should also be interpreted cautiously. This was a single-center study conducted at an urban tertiary referral hospital in Istanbul, and the cohort was predominantly male, clinically stable, and largely virologically suppressed, with limited representation of women and individuals with advanced HIV disease. Therefore, the findings may not fully generalize to women, gender-diverse individuals, PLHIV receiving care in rural areas or non-tertiary centers, or patients with more advanced disease. In addition, measurement invariance across key demographic and clinical subgroups was not evaluated. Because subgroup sizes were highly imbalanced, particularly by sex, formal invariance testing and subgroup-specific psychometric interpretation were not considered sufficiently powered. Similarly, the sensitivity power analysis should not be interpreted as confirmatory evidence of adequate power or validity; it only indicates the approximate effect size detectable with the available sample. Subgroup comparisons involving rare or highly imbalanced clinical categories, including history of AIDS-defining conditions, detectable viral load, and sex-based comparisons, should therefore be interpreted descriptively. Finally, tariff dependence remains a constraint until a national EQ-5D-5L value set becomes available (Tran, Ohinmaa & Nguyen, 2012; Popping et al., 2021). For future Turkish HIV studies, prespecifying a primary tariff with sensitivity analyses using alternative tariffs would improve comparability until a Turkey-specific EQ-5D-5L value set is developed.

## Conclusions

In adults with HIV receiving routine care, the Turkish EQ-5D-5L demonstrated acceptable measurement performance, with evidence supporting reliability and construct validity; CFA suggested a coherent internal response structure in this sample. From a practical perspective, the instrument appears suitable for group-level HRQoL profiling and between-group comparisons in HIV care. However, the substantial ceiling effect and wide EQ-VAS limits of agreement suggest that sensitivity to within-person change may be limited in clinically stable populations. Incorporating disease-specific measures as external criteria in future work would provide stronger convergent validity evidence (Cheng et al., 2024; Miners et al., 2014).

## Supplemental Information

10.7717/peerj.21727/supp-1 Supplemental Information 1 Inter-dimension Spearman correlation matrix (EQ-5D-5L) Spearman rank correlation matrix (ρ ) describing associations among the five EQ-5D-5L dimension responses at baseline (n=157). Values are Spearman’s ρ .

10.7717/peerj.21727/supp-2 Supplemental Information 2 Spearman correlation matrix among utilities, EQ-VAS, duration since diagnosis, and ART duration Spearman rank correlations (ρ ) among utility indices (by tariff), LSS, EQ-VAS, months since HIV diagnosis, and ART duration (n=157). Superscript “a” marks correlations with p ¡ 0.001.

10.7717/peerj.21727/supp-3 Supplemental Information 3 Internal consistency reliability indices for EQ-5D-5L dimensions Item-deletion and item–total statistics for the five EQ-5D-5L dimensions at baseline (n=157). “Cronbach’s *α* if item deleted” shows the scale alpha recalculated after removing each dimension; “corrected item–total correlation” is the correlation of each dimension with the sum of the remaining dimensions.

10.7717/peerj.21727/supp-4 Supplemental Information 4 Distribution of scores stratified by comorbidity, CD4 count, detectable viral load, and history of AIDS-defining condition Exploratory known-groups comparisons of utility indices (by tariff), LSS, and EQ-VAS across clinically defined subgroups with small or imbalanced sizes (history of an AIDS-defining condition; CD4 category; viral load detectability). Outcomes are median (Q1–Q3); p-values are from two-sided Mann–Whitney U tests; effect size is rank-biserial correlation (interpret direction in relation to the group order and scale direction: higher is better for utilities/EQ-VAS, higher is worse for LSS).

10.7717/peerj.21727/supp-5 Supplemental Information 5 The complete raw dataset De-identified participant-level raw dataset containing all variables used for the analyses

## Additional Information and Declarations

### Data Availability

The following information was supplied regarding data availability: The data is available in the Supplemental Files.
