# Supplementary material for: Reliability and validity of the Turkish EQ-5D-5L in adults living with HIV
Source: PeerJ. 2026 Sep 12;14:e21727. doi: 10.7717/peerj.21727 (PMC13576739; doi:10.7717/peerj.21727)
Supplement: Supplementary file 1 [file peerj-14-21727-s001.docx]

**Supplementary Table S1. Inter-dimension Spearman correlation matrix (EQ-5D-5L)**

| Item | Mobility | Self- care | Usual activities | Pain/ discomfort | Anxiety/ depression |
| --- | --- | --- | --- | --- | --- |
| Mobility | 1.000 | 0.576 | 0.713 | 0.633 | 0.388 |
| Self-care | 0.576 | 1.000 | 0.599 | 0.486 | 0.312 |
| Usual activities | 0.713 | 0.599 | 1.000 | 0.755 | 0.441 |
| Pain/discomfort | 0.633 | 0.486 | 0.755 | 1.000 | 0.505 |
| Anxiety/depression | 0.388 | 0.312 | 0.441 | 0.505 | 1.000 |

Note. Values are Spearman’s ρ for baseline EQ-5D-5L dimensions.
