# Supplementary material for: Reliability and validity of the Turkish EQ-5D-5L in adults living with HIV
Source: PeerJ. 2026 Sep 12;14:e21727. doi: 10.7717/peerj.21727 (PMC13576739; doi:10.7717/peerj.21727)
Supplement: Supplementary file 2 [file peerj-14-21727-s002.docx]

**Supplementary Table S2. Spearman correlation matrix among utilities, EQ-VAS, duration since diagnosis, and ART duration**

| Variable | UK 3L | Germany 5L | Hungary 5L | Egypt 5L | Morocco 5L | LSS | EQ-VAS | Duration Since Diagnosis | Duration of ART |
| --- | --- | --- | --- | --- | --- | --- | --- | --- | --- |
| UK 3L crosswalk | 1.000 | 0.983^a^ | 0.978^a^ | 0.965^a^ | 0.959^a^ | -0.973^a^ | 0.602^a^ | -0.060 | -0.060 |
| Germany 5L | 0.983^a^ | 1.000 | 0.994^a^ | 0.987^a^ | 0.988^a^ | -0.990^a^ | 0.608^a^ | -0.055 | -0.054 |
| Hungary 5L | 0.978^a^ | 0.994^a^ | 1.000 | 0.995^a^ | 0.993^a^ | -0.992^a^ | 0.615^a^ | -0.043 | -0.043 |
| Egypt 5L | 0.965^a^ | 0.987^a^ | 0.995^a^ | 1.000 | 0.994^a^ | -0.991^a^ | 0.609^a^ | -0.050 | -0.049 |
| Morocco 5L | 0.959^a^ | 0.988^a^ | 0.993^a^ | 0.994^a^ | 1.000 | -0.982^a^ | 0.610^a^ | -0.051 | -0.049 |
| LSS | -0.973^a^ | -0.990^a^ | -0.992^a^ | -0.991^a^ | -0.982^a^ | 1.000 | -0.614^a^ | 0.047 | 0.046 |
| EQ-VAS | 0.602^a^ | 0.608^a^ | 0.615^a^ | 0.609^a^ | 0.610^a^ | -0.614^a^ | 1.000 | -0.010 | -0.014 |
| Duration Since Diagnosis | -0.060 | -0.055 | -0.043 | -0.050 | -0.051 | 0.047 | -0.010 | 1.000 | 0.998^a^ |
| Duration of ART | -0.060 | -0.054 | -0.043 | -0.049 | -0.049 | 0.046 | -0.014 | 0.998^a^ | 1.000 |

Note. Values are Spearman’s ρ. Significance: ^a^ p < 0.001.
