# Supplementary material for: Reliability and validity of the Turkish EQ-5D-5L in adults living with HIV
Source: PeerJ. 2026 Sep 12;14:e21727. doi: 10.7717/peerj.21727 (PMC13576739; doi:10.7717/peerj.21727)
Supplement: Supplementary file 3 [file peerj-14-21727-s003.docx]

**Supplementary Table S3. Internal consistency reliability indices for EQ-5D-5L dimensions**

| Dimension | Cronbach’s α if item deleted | Corrected item–total correlation |
| --- | --- | --- |
| Mobility | **0.797** | **0.711** |
| Self-care | **0.826** | **0.621** |
| Usual activities | **0.771** | **0.808** |
| Pain/discomfort | **0.780** | **0.767** |
| Anxiety/depression | **0.885** | **0.491** |

Note. Cronbach’s α if item deleted indicates the overall scale reliability after removing the respective item; corrected item–total correlation represents the correlation between each item and the sum of the remaining items.
