# Supplementary material for: Reliability and validity of the Turkish EQ-5D-5L in adults living with HIV
Source: PeerJ. 2026 Sep 12;14:e21727. doi: 10.7717/peerj.21727 (PMC13576739; doi:10.7717/peerj.21727)
Supplement: Supplementary file 4 [file peerj-14-21727-s004.docx]

**Supplementary Table S4. Distribution of scores stratified by comorbidity, CD4 count, detectable viral load, and history of AIDS-defining condition**

| AIDS-defining condition | No History of AIDS-defining condition (n=152) | History of AIDS-defining condition (n=5) | p-value | Rank-biserial r |
| --- | --- | --- | --- | --- |
| UK 3L utility | 0.848 [0.689-1.000] | 0.639 [0.414-1.000] | 0.374 | 0.267 |
| Germany 5L utility | 0.970 [0.859-1.000] | 0.829 [0.756-1.000] | 0.414 | 0.250 |
| Hungary 5L utility | 0.960 [0.859-1.000] | 0.741 [0.739-1.000] | 0.331 | 0.289 |
| Egypt 5L utility | 0.946 [0.766-1.000] | 0.669 [0.607-1.000] | 0.341 | 0.284 |
| Morocco 5L utility | 0.964 [0.835-1.000] | 0.770 [0.661-1.000] | 0.379 | 0.266 |
| LSS | 6.0 [5.0-8.0] | 8.00 [5.0-11.0] | 0.436 | -0.161 |
| EQ-VAS | 88.00 [77.50-96.20] | 71.00 [70.00-92.00] | 0.358 | 0.282 |
| CD4 count | **>=500 (n=126)** | **<500 (n=31)** | **p-value** | **Rank-biserial r** |
| UK 3L utility | 0.848 [0.725-1.000] | 0.848 [0.568-1.000] | 0.434 | 0.342 |
| Germany 5L utility | 0.970 [0.882-1.000] | 0.918 [0.801-1.000] | 0.520 | 0.327 |
| Hungary 5L utility | 0.960 [0.876-1.000] | 0.907 [0.798-1.000] | 0.479 | 0.334 |
| Egypt 5L utility | 0.946 [0.780-1.000] | 0.840 [0.676-1.000] | 0.531 | 0.325 |
| Morocco 5L utility | 0.964 [0.841-1.000] | 0.894 [0.784-1.000] | 0.618 | 0.311 |
| LSS | 6.0 [5.0-8.0] | 7.00 [5.0-10.0] | 0.414 | -0.162 |
| EQ-VAS | 88.50 [78.25-95.00] | 84.00 [70.50-98.00] | 0.738 | 0.293 |
| Viral load | **Undetectable (n=148)** | **Detectable (n=9)** | **p-value** | **Rank-biserial r** |
| UK 3L utility | 0.848 [0.689-1.000] | 0.848 [0.414-1.000] | 0.444 | 0.215 |
| Germany 5L utility | 0.970 [0.851-1.000] | 0.918 [0.644-1.000] | 0.399 | 0.231 |
| Hungary 5L utility | 0.960 [0.851-1.000] | 0.907 [0.660-1.000] | 0.359 | 0.245 |
| Egypt 5L utility | 0.946 [0.766-1.000] | 0.842 [0.587-1.000] | 0.437 | 0.218 |
| Morocco 5L utility | 0.964 [0.835-1.000] | 0.899 [0.415-1.000] | 0.391 | 0.234 |
| LSS | 6.00 [5.0-8.0] | 7.00 [5.0-9.0] | 0.467 | -0.074 |
| EQ-VAS | 87.50 [75.75-95.00] | 91.00 [62.00-100.00] | 0.776 | 0.011 |

Note. Values are median [Q1-Q3]. Mann-Whitney U test (two-sided). Effect size is rank-biserial correlation. Positive values indicate higher scores in the first-listed group and negative values indicate higher scores in the second-listed group; interpretation should consider scale direction (higher is better for utilities and EQ-VAS; higher is worse for LSS).
